# Exploring the experiences of the Windrush Generation, living in UK care homes: protocol for a qualitative study using the Silences Framework

**DOI:** 10.1136/bmjopen-2025-109342

**Published:** 2026-02-19

**Authors:** Lorna Hollowood, Julie Taylor, Kerry Allen

**Affiliations:** 1 School of Nursing and Midwifery, University of Birmingham, Birmingham, UK; 2 School of Nursing and Midwifery, College of Medicine and Health, University of Birmingham, Birmingham, UK; 3 Birmingham Women’s and Children’s Hospital NHS Foundation Trust, Birmingham, UK; 4 Health Services Management Centre, University of Birmingham, Birmingham, UK

**Keywords:** Nursing Homes, Primary Health Care, QUALITATIVE RESEARCH, Aging, Vulnerable Populations

## Abstract

**Abstract:**

**Introduction:**

The Windrush Generation describes a group of individuals who migrated, primarily from the Caribbean to the UK between 1948 and 1971, many of whom are now entering older age. Now entering later life, many face ongoing health inequalities shaped by systemic racism and cultural marginalisation. Despite a growing number of ethnic minority residents in UK care homes, little is known about the lived experiences of Black African Caribbean people in these settings, particularly at the end of life.

**Methods and analysis:**

This qualitative study explores the experiences of Black African Caribbean care home residents and their families, focusing on how race, identity and marginalisation shape care. Guided by the Silences Framework, semistructured interviews will be conducted with up to 16 participants across diverse care home settings. Data will be analysed thematically, with attention to under-represented narratives. A Patient and Public Involvement group of African Caribbean community members has codeveloped the study and will support analysis and dissemination to ensure cultural relevance.

**Ethics and dissemination:**

Ethical approval has been secured (REC: 24/WM/0151; protocol number: RG_21087; IRAS project ID: 302629), and the study will follow rigorous consent and capacity procedures, including caregiver affirmation and UBACC assessment where needed. Given the sensitive, potentially distressing focus on racism, marginalisation and end-of-life experiences, the research will be conducted by an experienced clinician-researcher using a reflexive, ethically grounded approach that safeguards both participants and researcher. Interviews will be held in private, accessible settings with appropriate advocacy, safeguarding concerns will follow care home and national protocols, and all data will be securely stored, anonymised and managed under General Data Protection Regulation and university governance, with the University of Birmingham as sponsor and data controller.

**STRENGTHS AND LIMITATIONS OF THIS STUDY**

- Focus on under-researched population: addresses a significant gap in UK research on the experiences of Black African Caribbean residents in care homes.
- Use of the Silences Framework: provides a robust, critical lens to explore marginalised narratives often overlooked in mainstream health research.
- Culturally grounded design: codesigned with Black African Caribbean community members via a dedicated Patient and Public Involvement group, enhancing cultural relevance, trust and validity.
- Focused scope and recruitment challenges: while the study provides in-depth insights into the experiences of Black African Caribbean populations, its focus on one ethnic group and potential difficulties in recruiting participants from care home settings may limit the representativeness and broader applicability of findings to other minoritised communities.
- Contextual and sample limitations: the small, UK-based sample—though appropriate for qualitative research—may limit the generalisability of findings to other ethnic minority groups, care home settings or international contexts.

## Introduction

 The Windrush Generation refers to people who migrated from Caribbean countries to the UK between 1948 and 1971, many of whom arrived as children accompanying their parents. This migration began with the arrival of the HMT *Empire Windrush* in 1948, and those who came were invited to help rebuild postwar Britain, often working in sectors such as transport, manufacturing and the newly established National Health Service (NHS). Despite their vital contributions, many faced systemic racism, discrimination and marginalisation throughout their lives.^1^ Now entering later life, members of the Windrush Generation represent a growing proportion of the Black African Caribbean population in the UK, yet their specific needs—especially in care settings—remain poorly understood and under-researched.

Care homes are an integral part of the UK healthcare system, as sites which are providing care for older people with complex clinical needs.^2^ The growing care home population has increasing frailty, multimorbidity and dependency, and the sector delivers a whole person approach to care delivery. Trained staff and healthcare professionals manage long-term conditions, dementia and provide end-of-life care. They also provide social and emotional care, support families and contribute to hospital admission avoidance as part of the advance care planning processes.^3^

The need for high-quality, culturally competent care for older people from ethnic minority backgrounds is increasing, yet research exploring their lived experiences in care homes remains limited. Older people from ethnic minority backgrounds in the UK experience disproportionate health inequalities, which accumulate over time due to sustained racial discrimination and disadvantage.^4^ This includes disparities in palliative and end-of-life care provision, with evidence suggesting that these groups often face barriers to accessing services that meet their cultural needs.^5^ Recent studies^6^,^8^ have highlighted enduring health and social care inequities experienced by ethnic minority populations in the UK, particularly among those of Black African Caribbean descent. These disparities, exacerbated during the COVID-19 pandemic, are attributed to structural racism, socioeconomic deprivation and limited access to culturally sensitive healthcare services.^9^

Despite a growing population of older people in care homes—estimated at nearly half a million in the UK^10 11^—the perspectives of ethnic minority residents remain largely absent from the literature. Previous reviews^12^,^14^ have found a severe lack of UK-based studies focused on the experiences of ethnic minority groups in care homes, particularly regarding end-of-life care. This gap in research limits our understanding of how cultural needs are—or are not—met within care home settings.

A recent integrative review^14^ explored the needs of older people with dementia from culturally and linguistically diverse backgrounds living in residential aged care settings. The study synthesised findings from 15 qualitative, quantitative and mixed-methods studies and, as part of their key findings, highlighted culturally specific needs, such as language needs, access to traditional foods and social activities that maintain sense of identity for individuals. While this review reinforces the importance of culturally responsive care, it does not focus on the UK context or the unique experiences of Black African Caribbean people in UK care homes. Given the growing number of ethnic minority residents in UK care settings,^11^ this study, positioned at the intersection of health service delivery and health inequalities, addresses a silence or gap in how we understand how care is experienced by Black African Caribbean care home residents and their families. By exploring the provision of culturally responsive care in the care home sector, this study contributes to ongoing efforts to improve quality, safety and inclusivity in health service delivery for underserved populations.

These issues sit firmly within the medical and therapeutic scope of BMJ Open, particularly in relation to dementia, palliative care and systemic disparities in later life care.

### Aim and objective

#### Study aim

To explore the marginal discourses influencing the health and social care experiences of Black African Caribbean care home residents and their families in the UK, as expressed through their narratives.

#### Research question

What are the experiences of the Windrush Generation, of living in UK care homes?

#### Objectives

To explore how Black African Caribbean residents and their families experience the delivery of health and social care in UK care homes.

To explore how experiences of racism, discrimination and stereotyping, understood as forms of marginalised discourse, shape the lived experiences of Black African Caribbean residents and their families in UK care homes.

To investigate the experiences of access to care and the quality of that care for Black African Caribbean residents in UK care homes

To explore the role of communication and language barriers in the delivery of health and social care to Black African Caribbean residents in UK care homes.

To provide recommendations for improving the delivery of health and social care for Black African Caribbean residents in UK care homes based on the findings of the study.

## Methods and analysis

### Conceptual framework

**Figure 1:**
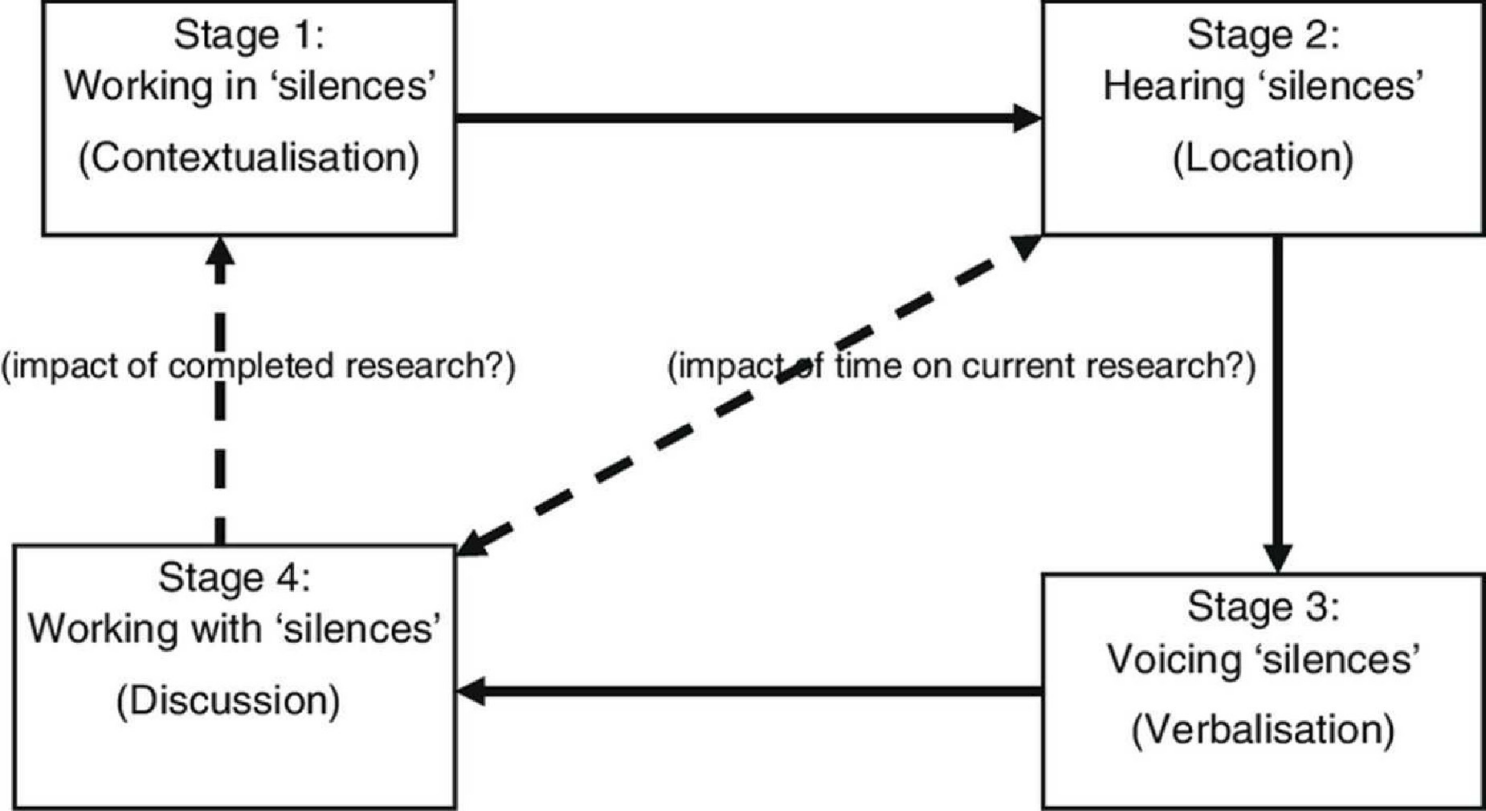
The Silences Framework.^15^

This qualitative study is underpinned by the Silences Framework,^15^ which highlights voices and experiences that are often absent or overlooked in mainstream healthcare research (figure 1). This theoretical framework is conceptualised as a basis for researching sensitive issues and the healthcare needs of those who belong to marginalised populations.^15^ ‘Silences’ refer to areas of research that are poorly understood, under-researched or, indeed, silenced, and the framework provides a process in which individuals’ interpretations of events and experiences can be valued and understood.^15 16^ The Silences Framework places emphasis on the personal experience and recognises the multiple perspectives in how we construct knowledge.^17^ The narratives collected by the residents and their carers will be analysed to answer the research question and address the objectives of the study. The Silences Framework proposes five stages that reflect the traditional research process, based on a criticalist philosophy, which will aid this study to uncover the perspectives of this group as well as serve as an advocate for their identified needs.^18^ Stage 1 primarily comprises identifying gaps and contextualising any silenced discourses through a systematic review of published research^19^ and a broader scope of grey literature and unpublished sources. The framework acknowledges that perspectives on experience of access and use of health and social care provision need to be interpreted on an individual basis. Marginalisation can occur where there is a heady combination of inequality and power imbalance, and we are keen as a research team not to use our position of power to further this discourse. Witt^20^ recognises that analysis that uncovers marginal discourse can reflect the position of the researcher. This potential for positionality bias will be mitigated by implementation of stage 2, by both regular reflexive practice from the researcher and the verification of the data by the Patient and Public Involvement (PPI) group, a group of elders who access an African Caribbean Centre that provides social care.

By applying this critical lens, the study will examine how care is experienced, accessed and understood by these communities, and identify ways to improve culturally competent care.

### Study design

This qualitative study has been designed and methodologically structured around the Silences’ Framework^15^ because of the way in which it was designed to open up a space for marginalised groups accessing healthcare. A qualitative descriptive approach using semistructured, in-depth interviews has been chosen to facilitate an in-depth exploration of participants’ experiences. This design is particularly suited to studies aiming to capture and present participants’ accounts in their own words, with minimal interpretation, making it well aligned with the study’s aim to inform practice and policy in a culturally sensitive and accessible manner. Semistructured interviews allow for a consistent yet flexible approach, enabling participants to share detailed narratives while allowing the interviewer to probe for deeper insights.^21^ To enhance cultural sensitivity and validity, interview questions have been codeveloped with Black African Caribbean community members,^22^ from a local Afro-Caribbean Centre who are collaborating as the community advisors throughout the study. Interviews will focus on participants’ experiences of ageing in the UK and navigating long-term care, from the initial decision-making process to transitions in care and end-of-life considerations (online supplemental table 1). Open-ended narrative prompts will be used to foster rapport and encourage storytelling.

### Participant selection

A purposive sampling approach will be used to recruit up to 16 participants, comprising residents and family carers or people identified as important to them. This inclusive approach allows the resident to identify someone they wish to be part of the conversation, if preferred, offering a source of support and a broader perspective on their experiences of living in a UK care home. This is in recognition of both the vulnerability of care home residents and acknowledges that telling stories and reminiscing with others is a robust and culturally affirming way of gathering and sharing knowledge.^23^ This sample size aligns with qualitative research guidance, which suggests that data saturation typically occurs within relatively homogeneous groups after approximately 12 interviews.^24^ The number of 16 participants allows for any extra people who contribute to the conversations. Data saturation and recruitment will guide final participant number. Participants will be eligible if they reside in long-term UK care settings, identify as Black African, Black Caribbean or of dual heritage (commonly associated with the Windrush Generation), and have the capacity to provide informed consent. Individuals will be excluded if they live independently, belong to other ethnic backgrounds, are unable or unwilling to participate, or are considered too unwell to engage. While statistical power calculations are not required for qualitative research, existing literature indicates that a sample of this size is sufficient to capture a diversity of experiences and allow for meaningful subgroup comparisons between residents and carers.^24^

### Data collection

This study will be set in UK care homes across multiple sites in the UK with identified residents who fit the inclusion criteria. The research team has worked in partnership with the NIHR Research Delivery Network, who support connections between researchers and care homes, to identify potential care homes to participate. Once a care home is identified as interested in participating in the study, the research team works with care home managers to identify individual residents who fit the study’s inclusion criteria, to be interviewed and conduct stage 3 of the Silences’ Framework; ‘Voicing silences’ to occur. Homes in diverse urban areas with known populations of people from the Windrush Generation will also be contacted for consideration. Participants will be identified by and accessed via lead social care providers, usually the registered care home manager of each home.

Interviews will be recorded and transcribed. Interviews will take place face to face wherever possible; however, it is recognised that this plan requires flexibility, particularly considering the vulnerability of residents living in the care home sector. Interviews may also take place virtually if that suits participants, healthcare settings or availability. While face-to-face interviews are preferred, virtual options (eg, Microsoft Teams, Zoom) will be provided to enhance accessibility.

### Data analysis

Stage 3 of the framework also aligns closely with the thematic analysis^25^ process. Coding and theme development will be employed to actively interrogate the narratives and consider where voices are heard or silenced in the data. A descriptive approach to analysis allows us to remain close to the data. This process will be informed and aligned with the Silences Framework,^15^ which provides a critical lens for uncovering marginalised, silenced or under-represented perspectives in healthcare research (table 1).

**Table 1:** Application of the Silences Framework^15^ in study design

Stage	Silences framework phase	Activities/methods
Stage 1	Working in ‘Silences’ (contextualisation)	Background and literature review(s)
Stage 2	Hearing ‘Silences’ (location)	Reflexive practice (researcher positionality). Patient and Public Involvement collaboration in developing data collection guide. Identification of philosophical underpinning
Stage 3	Voicing ‘Silences’ (verbalisation)	Data collection through 12–16 semistructured interviews with Black African Caribbean care home residents and their families. Thematic analysis and synthesis
Stage 4	Working with ‘Silences’ (discussion)	Critical discussion of findings. Researcher reflection
Stage 5	Planning for ‘Silences’ (recommendations)	Development of implications for health and social care policy, research and education

The Principal Investigator will lead the initial coding, with peer review by academic colleagues to enhance analytical rigour. A reflexive approach will be maintained throughout the analysis, guided by the principles of the Silences Framework^15^ to ensure sensitivity to structural inequalities and overlooked narratives. Insights from the PPI group will also be incorporated to support the credibility and cultural relevance of interpretations. NVivo software will be used to facilitate the coding process and data organisation. Member checking will be offered to participants to verify emerging themes and interpretations, further strengthening the trustworthiness of the findings.

Stage 4, ‘Working with silences’ will inform the interpretation and discussion of findings, ensuring that conclusions remain grounded in participants’ accounts while critically engaging with the structural and contextual factors that shape these experiences.

### Patient and public involvement (PPI)

PPI has been embedded throughout the development of this study to ensure cultural relevance and enhance the quality and impact of the research. A dedicated PPI group, comprising individuals from Black African Caribbean backgrounds with lived experience of receiving social care or supporting someone who has, contributed to the design of the study, including shaping the research questions, refining the interview topic guide and advising on recruitment strategies. Their input has been instrumental in ensuring that the study is respectful, inclusive and responsive to community concerns. The PPI group will continue to be actively involved during data interpretation, helping to validate emerging themes and ensuring that the findings are grounded in the lived realities of the populations under study. Members will also be engaged in dissemination planning, to help ensure that study outputs are accessible and impactful for relevant communities and stakeholders.

### Ethics and dissemination

Ethical approval has been obtained by the Coventry and Warwick Research Ethics Committee. REC reference: 24/WM/0151; protocol number: RG_21087; IRAS project ID: 302 629. Participants will be provided with detailed information sheets, and informed consent will be obtained prior to participation. Participant capacity will be assessed and affirmed by the primary caregiver in the care home. If concerns are raised during the interview process about fluctuation of capacity, an in-the-moment assessment will take place using the University of California Brief Assessment of Capacity to Consent (UBACC).^26^ If necessary, a designated proxy may assist in the decision-making process. Participants may withdraw from the data collection stage without repercussions.

It is important to recognise that this research will include conversations about sensitive issues with vulnerable participants. This presents important ethical issues and potential burden for the participants. The interviews will be carried out by a researcher with experience of discussing sensitive issues and with clinical expertise in caring for older people and their families. Given the sensitive and potentially distressing nature of the research, which involves exploring racism, marginalisation and end-of-life experiences, particular attention will also be paid to researcher well-being. Research on sensitive topics can expose researchers to emotionally challenging data, yet the psychological impact of this work is often overlooked in ethics processes and institutional support systems.^27^ As such, this study will adopt a reflexive and ethically grounded approach that recognises the emotional labour involved in conducting research with marginalised groups and aims to safeguard the researcher through supervision, debriefing and structured reflection.

Interviews will be conducted in private, secure and accessible settings within the care home or an alternative location agreed on by the participant, to ensure comfort, dignity and confidentiality. Where appropriate, carers or advocates may be present to support the resident’s participation without influencing their responses. Any safeguarding concerns arising during the research process will be managed in line with the host care home’s safeguarding protocols and national adult safeguarding legislation, including the Care Act 2014.^28^ The Principal Investigator will report concerns promptly to the designated safeguarding lead. All data will be handled in accordance with the university’s research governance policies, the General Data Protection Regulation (GDPR),^29^ and the Data Protection Act 2018.^30^ Audio recordings, transcripts and related materials will be securely stored on encrypted, password-protected university systems and anonymised to protect participants’ identities. The University of Birmingham is the nominated sponsor and data controller for this study.

### Outputs

This study aims to generate actionable insights to inform health and social care policy, applying stage 5 of the framework; ‘Planning for Silences’. These insights will enhance professional education and promote culturally competent and equitable care for Black African Caribbean people in UK care homes. Findings will be disseminated through multiple channels to maximise reach and impact. These will include publication in peer-reviewed academic journals, presentations at national and international conferences focused on adult social care, palliative care and health inequalities, and stakeholder engagement events. Tailored outputs will be developed for different audiences, including policy briefings for health and social care commissioners, guidance documents for care home managers and staff and accessible summaries for participant communities. Engagement with professional networks and advisory groups will further support the integration of findings into practice and policy development.

This study will contribute to policy discussions and clinical practice improvements by:

- Highlighting the lived experiences of Black African Caribbean people in UK care homes.
- Identifying systemic and cultural barriers in care provision.
- Offering evidence-based recommendations for care providers, policymakers and researchers to enhance cultural responsiveness in social care.

## Supplementary material

10.1136/bmjopen-2025-109342 online supplemental table 1
