# Supplementary material for: Exploring the experiences of the Windrush Generation, living in UK care homes: protocol for a qualitative study using the Silences Framework
Source: BMJ Open. 2026 Feb 19;16(2):e109342. doi: 10.1136/bmjopen-2025-109342 (PMC12927383; doi:10.1136/bmjopen-2025-109342)
Supplement: online supplemental table 1 [file bmjopen-16-2-s001.pdf]

Interview Guide

The experiences of the Windrush Generation of UK care homes – a qualitative study

The focus of the interview will be the care experiences of people living in care homes. The aim of the interview is to allow the participants to tell their stories in their own words.

The interview will start by building a rapport and will start with introductions and an explanation about the study.

Open ended questions will be asked and the conversation will have freedom to go where the participant wishes. As responses emerge the interview will be tailored to the answers given.

Suggested prompt questions

1. Introduction and demographic information

Can you tell me a little about yourself?

Can you tell me about your life before you came to the UK?

Can you tell me about your life in the UK?

Can you tell me about your family? Work?

Can you tell me about retiring and getting older?

2. Care home experiences

Can you tell me about living here in the care home? How long have you lived here?

How did you come to live in a care home, and what was the process like?

How did you decide which care home and who helped you?

How would you describe the quality of care that you have experienced in the care home?

Have you had any positive or negative experiences with care staff in the care home?

Have you had any positive or negative experiences with other residents in the care home?

How has living in the care home affected your physical or mental health?

Can you tell me about any challenges you have faced in the care home?

How have you found access to healthcare services since living in the care home?

Have you been able to participate in social activities?

Can you tell me about living here? Can you describe your care experiences?

What do you most enjoy about living in the care home? Could anything be improved?

How do you see your future in the care home, and what are your hopes and concerns?

Is there anything else you would like to share about your care home experiences?

3. Close and debrief

Check wellbeing and comfort, offer signposting to support if required. The National Advocacy service POhWER are accessed here https://www.pohwer.net/

Thank the participant for their time today
