# Supplementary material for: Exploring the experiences of the Windrush Generation, living in UK care homes: protocol for a qualitative study using the Silences Framework
Source: BMJ Open. 2026 Feb 19;16(2):e109342. doi: 10.1136/bmjopen-2025-109342 (PMC12927383; doi:10.1136/bmjopen-2025-109342)
Supplement: Supplementary file 2 [file bmjopen-2025-109342.reviewer_comments.pdf]

PEER REVIEW HISTORY

BMJ Open publishes all reviews undertaken for accepted manuscripts. Reviewers are asked to complete a checklist review form and are provided with free text boxes to elaborate on their assessment. These free text comments are reproduced below.

ARTICLE DETAILS

Title (Provisional)

Exploring the experiences of the Windrush Generation, living in UK care homes: protocol for a qualitative study using the Silences Framework

Authors

Hollowood, Lorna; Taylor, Julie; Allen, Kerry

VERSION 1 - REVIEW

Reviewer **1**

Name **Umubyeyi, Benoite**

Affiliation **Maison Médicale Jeanne Garnier, Département Recherche, Enseignement et Formation**

Date **26-Sep-2025**

COI **None**

Thank you for the opportunity to review this interesting protocol. This study addresses an important gap in understanding the lived experiences of older adults of Black African Caribbean heritage accessing palliative and end-of-life care. Below are my comments and suggestions to further strengthen the manuscript.

General comments

1. The use of “living and dying….” In the title is potentially ambiguous. This sounds as though the authors would recruit residents in the “dying phase”. If the aim of the study is to explore broader experiences of accessing palliative and end-of-life care of this group of individuals, and the factors that shape their care pathway, I would suggest avoiding using the “dying” in the title and objective of the study.

2. The phrasing of “older Black African Caribbean care home residents” appears somewhat reductive; it puts participants into a category rather than centering them as people first. Authors may consider reframing this term and keep consistency of terms used across the manuscript.

3. Page 4; lines 58-60: The sentence beginning “A recent integrative review…” reads as incomplete. Please add the missing verb.

4. Page 5, lines 54-57: The sentence "This will be mitigated by "implementation of Silences" is difficult to follow. It would help if the authors could briefly define what "Silences" refers to and how it will be applied in the study. As written, it is not obvious to the reader how and why "Silences" mitigates the influence of the researcher's positionality.

Aims and objectives

5. While the authors have clearly stated the study objectives, given the exploratory nature of the research aim and design, it would strengthen the manuscript to include specific research questions that the study addresses.

Study design and methods

6. While the proposed qualitative descriptive design is useful for providing narrative accounts of experiences, given the focus on lived experiences of accessing palliative care, a qualitative phenomenological approach might offer a deeper exploration of meaning and subjectivity. I would encourage the authors to reflect on whether phenomenology may be more aligned with their research aim, or alternatively, to clarify why a descriptive design is preferable in this context.

7. The authors intend to recruit up to 16 participants (10 residents and 6 family carers) using purposive sampling. Could they justify why these specific numbers and composition? It is not clear whether the six family carers are related to the participating residents and how they would be recruited in the study.

8. "This study will be set in UK care home across multiple sites within diverse areas of the UK": It is not clear which and how care homes will be selected, and what "diverse areas" refers to- (geographically? culturally?). It would be helpful for the authors to specify which care home sites will participate in the study or at least a clear selection strategy of sites rather than remaining vague.

9. The authors are recommended to include a draft interview guide or a sample of questions that will be asked. Will the same questions be asked to residents and to family caregivers?

10. The Silences Framework is identified as guiding the thematic analysis. However, it is not clear how the stages of the framework (particularly stages 1, 2, and 4) align with thematic analysis as presented in Table 1. According to the reference provided (Serrant-Green, 2011), phases 1 and 2 focus on identifying gaps and contextualizing silenced discourses from the literature, which may not directly involve the analysis of empirical data, while phase 4 relates to discussion and conclusion. Will phases 1 and 2 be addressed prior to data collection as part of the literature review and contextualisation process? I recommend elaborating further to explain how these phases will be applied to the analytical process within the study design. I also encourage the authors to engage with the analytical process suggested in phase 3 (Serrant-Green, 2011) to clarify how they intend to conduct data thematic analysis.

VERSION 1 - AUTHOR RESPONSE

Review Section	Revision suggested	Action Taken
1. Title	The use of "living and dying…." In the title is potentially ambiguous. This sounds as though the authors would recruit residents in the "dying phase". If the aim of the study is to explore broader experiences of accessing palliative and end-of-life care of this group of individuals, and the factors that shape their care pathway, I would suggest avoiding using the "dying" in the title and objective of the study.	Thank you for your suggestion, which has stimulated great discussion. Whilst I do not wish to mislead at all, life expectancy in UK care homes is around 11-18 months, which means that palliative and end of life care are core business in this sector. Transition into a care home is often seen as the final chapter, with a focus on people living well until they die. For that reason, I would like to keep dying in the title to enhance the recognition of this.<br>Of course I am happy to discuss further.
2.	The phrasing of "older Black African Caribbean care home residents" appears somewhat reductive; it puts participants into a category rather than centering them as people first. Authors may consider reframing this term and keep consistency of terms used across the manuscript.	Thank you for highlighting the importance of person-centered language. We agree that the suggested phrasing could appear reductive. In response, we have revised this terminology throughout the manuscript to use people-first language. For example, we now refer to participants as "Black African Caribbean people who live in care homes". Of note, and my rationale for including ethnicity first – is by request of my PPI group, who's preferred terminology is 'Black people'.<br><br>I am happy to discuss this further or add a

		terminology note to this protocol.
3.	Page 4; lines 58-60: The sentence beginning “review A recent integrative …” reads as incomplete. Please add the missing verb.	Thank you for this revision – sentence revised accordingly.
4.	Page 5, lines 54-57: The sentence “This will be mitigated by “implementation of Silences” is difficult to follow. It would help if the authors could briefly define what “Silences” refers to and how it will be applied in the study. As written, it is not obvious to the reader how and why “Silences” mitigates the influence of the researcher’s positionality.	You will note in the preceding paragraph I have now added the detail about the Silences Framework and rationale for choosing it which provides a better context for this commentary at the end. I really hope this provides the clarity needed.
5. Aims and objectives	While the authors have clearly stated the study objectives, given the exploratory nature of the research aim and design, it would strengthen the manuscript to include specific research questions that the study addresses.	Thank you for your helpful suggestion. In response, we have now included the full aims and objectives of the study in the revised manuscript.
6. Study design and methods	While the proposed qualitative descriptive design is useful for providing narrative accounts of experiences, given the focus on lived experiences of accessing palliative care, a qualitative phenomenological approach might offer a deeper exploration of meaning and subjectivity. I would encourage the authors to reflect on whether phenomenology may be more aligned with their research aim, or alternatively, to clarify why a descriptive design is preferable in this context.	Thank you for your thoughtful comment and suggestion. We have carefully considered a range of qualitative approaches during the design phase.<br>Our decision to adopt a qualitative descriptive design has been guided by the Silences Framework and the specific objectives of the study, which focus on capturing and presenting participants’ accounts in a way that remains close to their own words and experiences.<br>While phenomenology offers a valuable lens for exploring meaning and subjectivity, our aim is not

		to generate interpretive theory or deeply abstracted essence but rather to provide a rich, straightforward description of experiences within their social and structural context. Qualitative descriptive design is particularly suited to studies where practical implications for policy and practice are central, and where accessibility of findings for diverse audiences, is a priority.<br>The Silences Framework complements this approach by emphasising the amplification of marginalised voices and avoiding over-interpretation, ensuring that participants' narratives remain authentic and grounded in their lived realities.<br>We appreciate your recommendation and will clarify this rationale in the manuscript to ensure alignment between our research aim and methodological choice.
7.	The authors intend to recruit up to 16 participants (10 residents and 6 family carers) using purposive sampling. Could they justify why these specific numbers and composition? It is not clear whether the six family carers are related to the participating residents and how they would be recruited in the study.	This information has now been included and will hopefully explain that by supporting residents to participate in the research by allowing a family member to join if they wish, enhances both the the inclusivity and the culturally affirming approach adopted.

8.	"This study will be set in UK care home across multiple sites within diverse areas of the UK": It is not clear which and how care homes will be selected, and what "diverse areas" refers to- (geographically? culturally?). It would be helpful for the authors to specify which care home sites will participate in the study or at least a clear selection strategy of sites rather than remaining vague.	Thank you for pointing out the lack of clarity here, we have revised the section and hopefully how care homes are being identified is much clearer now.
9.	The authors are recommended to include a draft interview guide or a sample of questions that will be asked. Will the same questions be asked to residents and to family caregivers?	Thanks – I have included some sample questions as a supplementary file.
10.	The Silences Framework is identified as guiding the thematic analysis. However, it is not clear how the stages of the framework (particularly stages 1, 2, and 4) align with thematic analysis as presented in Table 1. According to the reference provided (Serrant-Green, 2011), phases 1 and 2 focus on identifying gaps and contextualizing silenced discourses from the literature, which may not directly involve the analysis of empirical data, while phase 4 relates to discussion **and** conclusion. -I recommend elaborating further to explain how these phases will be applied to the analytical process within the study design. I also encourage the authors to engage with the analytical process suggested in phase 3 (Serrant-Green, 2011) to clarify how they intend to conduct data thematic analysis.	Thank you for this helpful feedback. We have now more explicitly mapped the application of the Silences Framework throughout the study design. In the revised manuscript, we clarify how phases 1 and 2 informed the development of our analytical lens and coding framework. We also elaborate on the integration of phase 3 with our thematic analysis process. Finally, we explain how phase 4 will guide interpretation and discussion.

VERSION 2 - REVIEW

Reviewer 1

Name **Umubyeyi, Benoite**

Affiliation **Maison Médicale Jeanne Garnier, Département Recherche, Enseignement et Formation**

Date **08-Dec-2025**

COI

The authors have addressed most of my previous suggestions, and I appreciate the revisions made.

One concern remains regarding the use of the term “dying” in the title. Although I understand the authors’ preference to retain it, I would encourage them to consider whether the current manuscript sufficiently engages with the concepts of living and dying in care homes. As it stands, the focus appears to be more on accessing care (which may include palliative and end-of-life care) rather than on the experiences of living and dying. It may therefore be helpful either to elaborate on the rationale for specifying the concept in the title while the rest of the manuscript only briefly mention it, or to broaden the title so that it more accurately reflects the manuscript’s emphasis on residents’ experiences of receiving care and end-of life care in these settings.

Research question: Please rephrase the sentence for clarity. As currently written, it appears that a word may be missing.

Aim of the study: I recommend revisiting the phrasing of the research aim. The current aim: “to analyse the narratives of…” reads more like a description of the qualitative method rather than a clearly defined research objective. Reframing the aim to emphasise the phenomenon under exploration (e.g., retaining the latter part beginning with “to explore…”) would strengthen the clarity and focus of the protocol.

Same observation for objective number 1. I would recommend keeping objective number 3 instead.

VERSION 2 - AUTHOR RESPONSE

**Review Section**	**Revision suggested**	**Action Taken**
**Revisions Jan 26**		
Title	One concern remains regarding the use of the term “dying” in the title.	Dying has been omitted from the title.

Research question:	Please rephrase the sentence for clarity. As currently written, it appears that a word may be missing.	Word added, thanks for spotting.
Aim of the study:	I recommend revisiting the phrasing of the research aim. The current aim: "to analyse the narratives of…" reads more like a description of the qualitative method rather than a clearly defined research objective. Reframing the aim to emphasise the phenomenon under exploration (e.g., retaining the latter part beginning with "to explore…") would strengthen the clarity and focus of the protocol.	I have revised the aim in line with the advice – thank you, hopefully sounds a bit clearer now.
Study objectives	Same observation for objective number 1. I would recommend keeping objective number 3 instead.	I have revised the objective number in line with advice provided – thank you.
